# Cancer Treatment–Induced Bone Loss (CTIBL): State of the Art and Proper Management in Breast Cancer Patients on Endocrine Therapy

**DOI:** 10.1007/s11864-021-00835-2

**Published:** 2021-04-16

**Authors:** Anna Diana, Francesca Carlino, Emilio Francesco Giunta, Elisena Franzese, Luigi Pio Guerrera, Vincenzo Di Lauro, Fortunato Ciardiello, Bruno Daniele, Michele Orditura

**Affiliations:** 1 Medical Oncology, Department of Precision Medicine, School of Medicine, “Luigi Vanvitelli” University of Campania, 80131 Naples, Italy; 2 Medical Oncology Unit, Ospedale del Mare, 80147 Naples, Italy; 3 Istituto Nazionale per lo Studio e la Cura dei Tumori, Fondazione Pascale, Istituto di Ricovero e Cura a Carattere Scientifico, Naples, Italy

**Keywords:** Breast cancer, Endocrine therapy, Bone mineral density, Fracture risk, Bisphosphonates, Denosumab

## Abstract

About 70–80% of early breast cancer (BC) patients receive adjuvant endocrine therapy (ET) for at least 5 years. ET includes in the majority of cases the use of aromatase inhibitors, as upfront or switch strategy, that lead to impaired bone health. Given the high incidence and also the high prevalence of BC, cancer treatment–induced bone loss (CTIBL) represents the most common long-term adverse event experimented by patients with hormone receptor positive tumours. CTIBL is responsible for osteoporosis occurrence and, as a consequence, fragility fractures that may negatively affect quality of life and survival expectancy. As recommended by main international guidelines, BC women on aromatase inhibitors should be carefully assessed for their fracture risk at baseline and periodically reassessed during adjuvant ET in order to early detect significant worsening in terms of bone health. Antiresorptive agents, together with adequate intake of calcium and vitamin D, should be administered in BC patients during all course of ET, especially in those at high risk of osteoporotic fractures, as calculated by tools available for clinicians. Bisphosphonates, such as zoledronate or pamidronate, and anti-RANKL antibody, denosumab, are the two classes of antiresorptive drugs used in clinical practice with similar efficacy in preventing bone loss induced by aromatase inhibitor therapy. The choice between them, in the absence of direct comparison, should be based on patients’ preference and compliance; the different safety profile is mainly related to the route of administration, although both types of drugs are manageable with due care, since most of the adverse events are predictable and preventable. Despite advances in management of CTIBL, several issues such as the optimal time of starting antiresorptive agents and the duration of treatment remain unanswered. Future clinical trials as well as increased awareness of bone health are needed to improve prevention, assessment and treatment of CTIBL in these long-term survivor patients.

## Introduction

Cancer treatment–induced bone loss (CTIBL) is considered the most common long-term adverse event experienced by breast cancer patients. Breast cancer (BC), which is the most frequent tumour in women worldwide, regardless of age, is characterized by a peak of incidence in postmenopausal age (50–69 years) [1]. Given the high incidence and also the high prevalence of long survivors, growing attention is now focused on the long-term effects of therapies that may negatively affect the quality of life of BC patients [2]. In this setting, decrease in bone mineral density (BMD) is mainly related to two factors, namely hypogonadism onset due to chemotherapy or gonadotropin-releasing hormone agonist (GnRH agonist) and endocrine therapies, which, added to menopausal related bone loss, are responsible for osteopenia or osteoporosis occurrence and, as a consequence, fragility fractures resulting in hospitalizations, disability and, sometimes, higher risk of mortality [3, 4]. In this review, we focus on physiopathology, diagnosis and management of CTIBL in BC.

## Physiology of bone turnover and pathogenesis of bone loss

Bone undergoes a lifelong physiological remodelling which is responsible for preserving bone integrity and its mineral mass as well as for maintaining mineral homeostasis. This active and dynamic process represents the result of the correct balance between sequential bone resorption by osteoclasts and bone deposition by osteoblasts at the same spatial location [5]. These two processes must be tightly coupled quantitatively, as well as in time and space, in order to assure the proper maintenance of skeletal functions. When the coupling is lost, the process is impaired and bone mass could be altered: an increase in resorption, as it happens during menopause, or a decrease in bone formation, as it happens with increasing age, may lead to several skeletal diseases, including osteoporosis [6, 7].

Peak bone mass, generally defined as the amount of bone tissue present at the end of the skeletal maturation, typically occurs at the beginning of the adulthood, with a predicted median age for females in early 30s [8]. Its achievement relies upon multiple concomitant factors, including genetic factors, physical activity, strength, diet, smoking habit, alcohol intake and hormonal status [9]. After the peak is reached, a slight decrease in bone formation occurs at each remodelling cycle, with different annual bone loss rates according to sex and, for women, menopausal status [10]. This physiological mechanism is the result of the interplay among age-related decrease in osteoblast differentiation and replication, reduced levels of local and systemic growth factors and age-related hypogonadism [5].

In particular, an annual decrease in BMD of 2% has been estimated during the first 10 years after the menopause [11]. A pivotal role in post-menopausal bone loss is surely played by the physiological decrease in the circulating estrogen levels since it is related to the release of several pro-inflammatory cytokines (IL-1, IL-6, TNF-α) by circulating monocytes and bone marrow cells [12, 13]. This plethora of soluble factors is responsible for recruiting osteoclast precursors thus inducing their differentiation as well as enhancing their activity by increasing the levels of RANK ligand (RANKL) and decreasing osteoprotegerin (OPG) levels [14]. Despite being coupled with a compensatory bone formation, resorption typically overcomes synthesis thus resulting in bone loss.

Noteworthy, the RANKL/RANK/OPG axis is likely to be involved in the antiresorptive action of estrogens since it plays a key role in bone remodelling through downstream NFK-B and Jun N-terminal kinases pathways. Finally, in vitro studies have demonstrated that estrogens may upregulate gene expression of OPG as well as may suppress RANKL expression in bone-lining cells. As a consequence, their lack may result in a significant impairing of this essential axis [15].

## Bone-related effects of BC adjuvant treatments

Adjuvant chemotherapy could cause bone loss as a consequence of ovarian failure in premenopausal women [16•] and, presumably, through estrogen independent mechanisms [17]. However, the greater negative effect on bone tissue in the adjuvant setting, both for a more specific impact on bone metabolism and for the longer time of administration, is related to endocrine treatment.

Endocrine therapy (ET) represents the standard of care in the adjuvant setting as it showed to significantly reduce the risk of recurrence and cancer-related death in hormone receptors positive (HR+) BC patients, which represent about 75–80% of all BC diagnoses [18•].

Adjuvant ET consists of two main drug classes: estrogen receptor modulators (tamoxifen) and aromatase inhibitors (anastrozole, letrozole, exemestane). Tamoxifen is a selective estrogen receptor modulator (SERM) [19] which exerts a different behaviour on bone tissue according to menopausal status: in postmenopausal women, it typically shows a pro-estrogenic effect on bone thus increasing bone density and reducing bone resorption, while in premenopausal women it induces a severe bone loss [20, 21]. Aromatase inhibitors (AIs), divided into non-steroidal (anastrozole and letrozole) and steroidal (exemestane) reduce biosynthesis of estrogens, causing an annual bone loss of 2.2–2.6% at lumbar spine and 1.7–2.1% at hips [22, 23]. Notably, recent studies have focused on potential genetic mechanisms which could be responsible for predisposing patients to AI-related osteoporosis. Particularly, at a genome wide analysis, three single nucleotide polymorphisms (SNPs) have been identified in 6 genes located at chromosome 20 (CTSZ, SLMO2, ATP5E), 6 (TRAM2, TRAM14A) and chromosome 2 (MAP4K4) which seemed to be significantly related to a higher fracture risk in women on AIs; preclinical studies suggest that the expression of these 6 genes is modulated by estrogens and their downregulation during estrogen suppression is related to upregulation of genes which promote osteoporosis [24].

## CTIBL in adjuvant ET trials

Most of data on CTIBL related to adjuvant ET administration derive from several randomized studies on ET where bone loss was reported as an adverse event, as well as from sub-analyses conducted on the same trials in order to determine the changes in BMD and in the risk of bone fractures (Table 1).

**Table 1:** Incidence of bone fractures in clinical trials with aromatase inhibitors in postmenopausal HR+ early BC patients

Clinical trial (n. of pts. enrolled)	Experimental arm	Control arm	Primary endpoint	Bone fracture incidence
ATAC (6241)	ANA	TAM	DFS: HR 0.85 in favor of ANA *p* = 0.003	Annual rate: 2.9% vs 1.9% *p* < 0.0001
BIG 1–98 (8010)	LETRO	TAM	DFS: HR 0.81 in favor of LETRO *p* = 0.003	Overall incidence: 9.3% vs 6.5% *p* = 0.002
MA-17 (5157)	LETRO	PLC	4y-DFS: 93% vs 87% *p* < 0.001	Overall incidence: 3.6% vs 2.9% *P* = 0.24
IES (4742)	TAM- > EXE	TAM	4y-DFS: 93% vs 87% *p* < 0.001	Overall incidence: 7% vs 5% *p* = 0.003

*ANA*, anastrozole; *DFS*, disease free survival; *LETRO*, letrozole; *PLC*, placebo; *TAM*, tamoxifen; *EXE*, exemestane

Non-steroidal AIs, letrozole and anastrozole, as adjuvant therapy in HR+ postmenopausal women, seem to exert a similar effect on bone tissue, even in the absence of a direct comparison.

Letrozole given for 5 years determined a higher risk of bone fractures with respect to tamoxifen (9.3% vs 6.5%, respectively) in BIG 1-98 trial [25]; however, notably, MA.17 trial demonstrated that the use of letrozole in the extended adjuvant setting (5 years of letrozole after the completion of standard 5 years of tamoxifen), despite significantly increasing disease-free survival (DFS), did not result in a statistically significant higher rate of clinical fractures (3.6% vs 2.9%, *p*=0.24) as well as incidence of newly diagnoses osteoporosis (5.8% vs 4.5%, *p*=0.07) compared to placebo group, suggesting that the major effect on bone loss is restricted to the first 5 years of adjuvant treatment [26].

A significant increased risk of bone fractures was recorded during 5 years of adjuvant anastrozole therapy with respect to tamoxifen, with a yearly fracture episode rate of 2.9% vs 1.9%, in the ATAC (Arimidex, Tamoxifen, Alone or in Combination) trial [27]. The effect of anastrozole on BMD beyond its scheduled suspension after 5 years has been investigated in a sub-protocol of the same trial, reporting a recovery in lumbar spine BMD (+2.4% and +4.0% at years 6 and 7, respectively) and the absence of further loss at the hip (+0.7% and +0.5%, respectively) [28].

Furthermore, the IES trial compared 3–2 years of tamoxifen followed by 2–3 years of exemestane (switch strategy) versus tamoxifen alone for the full 5 years. Interestingly, a non-statistically significant increase of incidence of fractures among patients who were randomized to switch strategy compared to those who continued tamoxifen for 5 years was reported (3.1% vs 2.3%, *p*=0.08) [29]. Moreover, in a sub-group analysis, a reduction in BMD both at the lumbar spine (−2.7%) and at the hip (−1.4%) was registered in women on exemestane within 6 months compared with baseline [30], but no difference in mean BMD changes from baseline to 24 months post end of treatment was observed between the two arms [31], highlighting the possibility of a complete revert in BMD after steroidal AI suspension.

Comparing non-steroidal AIs in FACE trial [32], in which postmenopausal HR+ early BC patients were randomized to receive letrozole or anastrozole, but also comparing steroidal and non-steroidal AIs in MA.27 trial [33], in which an analogue population was assigned to anastrozole or exemestane, no significant differences in BMD modification were reported, underlining a “class” effect on bone tissue, independently from the molecule. Of note, survival outcomes and safety profile were comparable among different AIs.

Finally, an Italian cross-sectional study explored the prevalence of vertebral fractures (VFs) before and during AI intake in HR+ postmenopausal early BC women: 263 patients were consecutively enrolled to receive dual energy X-ray absorptiometry to evaluate BMD and to identify VFs by quantitative morphometry. Surprisingly, a VFs prevalence of 31.2% and 18.9% in AI-therapy arm and in AI-naïve patients was registered, respectively. Interestingly, in the latter ones, bone damage was typically related to older age and BMD values at femoral neck, while in AI-treated group the prevalence of VFs was not significantly different between patients with osteoporosis and those with normal BMD, underlining an independency from mineralization before and during treatment [34•]. Moreover, fat body mass has been shown to be associated to higher risk of VFs in AI-treated women, but not in naïve ones, in a similar population of study [35].

## Clinical assessment of fracture risk

Women affected by BC treated with AIs as adjuvant therapy or in premature iatrogenic menopause represent a peculiar subtype of patients at high risk for fracture, in which a careful assessment of risk is strongly recommended by the main international guidelines, including ASCO and ESMO ones [36•, 37•, 38].

Fracture risk is difficult to assess since several factors should be considered. Smoking history, alcohol consumption, physical activity, diet and family history exert a pivotal role in individualized clinical assessment of fracture risk [39]. Moreover, it must be noted that cutoff thresholds used to define advanced age, alcohol consumption and low body weight vary among the trials.

Dual-energy X-ray absorptiometry (DXA) represents a standard diagnostic tool in assessing BMD: through the analysis of lumbar spine and hip scans, measurements are interpreted using the WHO T-score definition of both osteopenia and osteoporosis [40]. DXA scans can be used at several levels: diagnosis of osteoporosis, fracture risk assessment and monitoring treatment response [41].

Even if a baseline and periodic assessment of BMD through DXA scans is strictly recommended in women at high risk of fracture, several studies suggested that this test is not enough by itself to estimate the risk of skeletal events, since other factors should be considered. Moreover, it has been showed that BMD evaluated through DXA scans can be normal or just slightly decreased in CTIBL even in the presence of VFs identified by a quantitative morphometric approach [34•].

A real-life population study highlighted disparities in BMD testing among patients on ET with AIs, with a lower compliance in case of older age and comorbidities [42]; furthermore, a retrospective analysis confirmed that older age was associated with absence of baseline DXA scan [43•].

To date, there is a lack of consensus about the BMD threshold to adopt in order to start a pharmacological intervention. BMD T-score diagnostic thresholds have been sometimes used even as a cut-off for administering antiresorptive drugs [44], but this approach is no longer acceptable. In the latest years, a growing number of consensus and expert opinions have proposed to prescribe antiresorptive drugs even with low BMD values.

The FRAX® tool, one of the most used algorithms available online which was developed to estimate the 10-year risk of a major osteoporotic fracture (spine, hip, humerus or wrist), can be used in combination with BMD assessment to identify patients with osteopenia and those with major risk of fracture, who could be eligible for treatment with bone-modifying agents [45, 46]. The FRAX® score is assessed by integrating in an algorithm model several dichotomous variables such as age, BMI, smoking habit, personal and familial history of fracture, glucocorticoid administration and presence of risk factors for secondary osteoporosis. However, besides its intrinsic limitations, FRAX score was not designed specifically to evaluate the fracture risk in BC women; thus, it could underestimate, or even overestimate, the effect on bone tissue of AI-therapy, depending on its inclusion as secondary osteoporosis cause or not in the algorithm [47•].

In order to overcome these limitations and improve prognostic accuracy, new useful tools have been developed, such as the trabecular bone score (TBS) [48]. TBS is a new analytical method based upon DXA image able to evaluate the bone microarchitecture providing skeletal information that is not captured from the standard BMD measurement. Interestingly, TBS is able to detect differences between DXA scans that show similar BMD measurements; particularly, it has been demonstrated to predict osteoporotic fractures regardless of bone density and to be independently associated with fracture risk. Moreover, TBS values are influenced by antiresorptive therapies with different magnitudes, possibly suggesting the use of this score as new outcome for bone mineralization in clinical trials [48, 49].

Finally, biochemical biomarkers of bone turnover (namely: alkaline phosphatase, hydroxyproline, C-terminal crosslinked telopeptide of type collagen I), despite great potential and ongoing evaluation, to date are not routinely used in the fracture risk assessment due to their intrinsic intra- and inter-individual variability [50].

Bone biomarkers of both resorption and formation have been investigated in HR+ BC postmenopausal women receiving exemestane showing an increase from baseline during treatment [30]; these results were confirmed in a trial comparing exemestane and tamoxifen, reporting also a reduction in bone biomarker levels during tamoxifen treatment [51]. Bone biomarkers could probably better stratify BC patients into fracture risk categories, thus implementing prevention of skeletal related events in specific subsets of postmenopausal women undergoing AIs.

## CTIBL management

### Non-pharmacological interventions

Smoking habit is associated with an increased risk of osteoporosis, and the risk decreases with the duration of cessation [52, 53], while alcohol consumption seems to exert different effects on BMD according to drinking pattern and levels [54]. Coffee consumption was not significantly associated with neither femoral neck nor lumbar spine BMD, and previous reports of negative effects of caffeine should be ascribed to lower calcium intakes rather than direct consequence [55, 56].

Exercise is widely recommended to prevent and reduce osteoporosis, but also to decrease related fragility fractures and their causative factors such as falls [57].

### Calcium and vitamin D

Adequate calcium daily intake through diet is a therapeutic goal in postmenopausal BC patients on adjuvant ET. Preferred sources of calcium are milks and dairy products, and the recommended daily intake is 1200 mg of calcium for women aged >50 years [58]. Vitamin D, which is both produced by skin after exposure to UVB and taken through diet (cheese, egg yolk, fish), is converted into 25-hydroxyvitaminD (25-OHD) in the liver, then into 1,25-dihydroxyvitamin D (1,25(OH)2D) in the kidney under the effect of parathormone (PTH) [59]. Vitamin D insufficiency, which is often defined as plasma 25(OH)D <30 nmol/l, is frequent in older women, resulting in elevated levels of serum PTH (secondary hyperparathyroidism) and bone weakening [60]: in this case, oral supplementation (400–800 UI per day) should be started [61]. The NCCN guidelines recommend a daily oral intake of 1200 mg total calcium and 800–1000 IU vitamin D in women at high risk for developing CTIBL [62]. However, in postmenopausal BC patients on adjuvant ET, calcium and vitamin D supplementations alone are not enough to prevent and treat CTIBL, even if randomized clinical trials evaluating their role in this specific population are lacking [63]. To date, supplementation of calcium and vitamin D plays a synergistic role in association with antiresorptive drugs by reducing the fracture risk and preventing hypocalcemia [36•].

### Antiresorptive drugs and BMD

Among antiresorptive drugs, two main classes have been approved to date for clinical practice: bisphosphonates and denosumab.

Bisphosphonates, discovered in 1960s searching for more stable analogues of inorganic pyrophosphate with high affinity for bone mineral through binding hydroxyapatite crystals [64], inhibit osteoclast-mediated bone resorption as main pharmacological mechanism of action. Members of this pharmaceutical class differ in chemical structure, showing different potency in antiresorptive activity and bioavailability: they could be administered orally (alendronate, risedronate and ibandronate) or intravenously (ibandronate and zoledronate) [65]. Bisphosphonates have shown to increase BMD and reduce the risk of fractures, thus leading to their approval in osteoporosis prevention and treatment [66]. A meta-analysis evaluating their efficacy in preventing vertebral, hip, and nonvertebral-nonhip fractures showed a non-superimposable efficacy among the drugs. In particular, the reduction rate of vertebral and hip fractures was higher with zoledronate while a higher decrease of nonvertebral-nonhip fractures was reported with risedronate [67].

Concerning the role of bisphosphonates in postmenopausal BC patients treated with ET, first data were published in late 1990s: adding 1600 mg daily of oral clodronate to oral tamoxifen 20 mg daily or oral toremifene 60 mg daily resulted in higher BMD at the lumbar spine and at the hips, than those who did not receive it [68]; however, these results are anachronistic since the current adjuvant ET standard in postmenopausal BC patients is the administration of AIs.

ARIBONE trial demonstrated the favourable impact of ibandronate on BMD in postmenopausal women treated with anastrozole, with a gain of +2.98% at the lumbar spine and +0.60% at the hip after 2 years of treatment [69]. Concerning risedronate, in SABRE trial a significant increased BMD in both lumbar spine (+2.2%) and total hip (+1.8%) by adding oral risedronate to anastrozole was reported [70]. These results were confirmed by a similar study, IBIS-II trial [71].

Zoledronate is absolutely the most studied bisphosphonate in BC patients on adjuvant ET. In premenopausal women, the addition of zoledronate 4 mg, given intravenously every 6 months, to adjuvant ET (goserelin plus tamoxifen or anastrozole) resulted in increased BMD in both lumbar spine and hips after 60 months, as reported in ABCSG 12 trial [72]. Concerning postmenopausal women, the changes in bone marker measurements observed in a subset of patients receiving letrozole plus upfront versus delayed zoledronate suggest that zoledronic acid’s effect on bone remodelling is both rapid and sustained over at least 1 year [73]. MA.27B trial, which is the largest prospective bone study designed to evaluate the efficacy of oral bisphosphonate treatment in HR+ postmenopausal women on concomitant AIs (anastrozole vs exemestane), enrolled 300 patients with BMD T-scores (spine and hip) of more than −2.0 and 197 patients with at least one T-score less than −2.0. Both groups received vitamin D and calcium, but only in the second one, bisphosphonates were administered. This trial highlighted that osteoporosis could be easily managed in patients with lower BMD values independently from the AI used, with very rare occurrence of clinical fractures. Definitely, despite the likelihood of strong estrogen suppression during treatment with AIs, bisphosphonates are able to prevent AI-induced bone loss [74].

Three trials (Z-FAST, ZO-FAST and E-ZO-FAST) have compared an upfront strategy (bisphosphonate at the time of adjuvant AI start) with delayed strategy (bisphosphonate at the evidence of BMD loss or in case of non-traumatic fracture) [75–77]. Overall, these trials have demonstrated a significant benefit in BMD in the upfront arm compared to the delayed one, although this gain in BMD did not translate into a reduction of fracture risk (Table 2).

**Table 2:** Clinical trials evaluating bisphosphonates or denosumab for the prevention of CTIBL in postmenopausal HR+ early BC patient treated with aromatase inhibitors

Clinical trial (n. of pts. enrolled)	Experimental arm	Control arm	Follow-up	BMD variation (experimental vs control arm)
ARIBON (50)	ANA + IBR	ANA + PLC	2 years	- lumbar spine: +2.98% vs −3.22% (*p* < 0.001) - hip: +0.6% vs −3.9% (*p* < 0.001)
SABER (154)	ANA + RSD	ANA + PLC	2 years	- lumbar spine: +2.2% vs −1.8% (*p* < 0.0001) - hip: +1.8% vs −1.1% (*p* < 0.0001)
IBIS II (150)	ANA + RSD	ANA + PLC	3 years	- lumbar spine: +1.1% vs −2.6% (*p* < 0.0001) - hip: −0.7% vs −3.5% (*p* = 0.0001)
Z-FAST (602)	LETRO + UPF ZLD	LETRO + DEL ZLD	1 year	- lumbar spine: +8.9% in favor of UPF (*p* < 0.0001) - hip: +6.7% in favor of UPF (*p* < 0.0001)
ZO-FAST (1065)	LETRO + UPF ZLD	LETRO + DEL ZLD	5 years	- lumbar spine: +4.3% vs −5.4% (*p* < 0.0001) - hip: +1.6% vs −4.2% (*p* < 0.0001)
E-ZO-FAST (527)	LETRO + UPF ZLD	LETRO + DEL ZLD	1 year	- lumbar spine: +4.3% vs −5.4% (*p* < 0.0001) - hip: +1.6% vs −4.2% (*p* < 0.0001)
ABSG-18 (3420)	AI + DEN	AI + PLC	3 years	- lumbar spine: +7.27% vs −2.75% (*p* < 0.0001) - hip: +4.6% vs −3.32% (*p* < 0.0001)

*AI,* aromatase inhibitor; *ANA*, anastrozole; *BMD*, bone mineral density; *DEL*, delayed; *DEN*, denosumab, *HR+*, hormone receptor positive, *IBR*, ibandronate; *LETRO*, letrozole; *PLC*, placebo; *RSD*, risedronate, *TAM*, tamoxifen; *UPF*, upfront, *ZLD*, zoledronate

Denosumab is an IgG2 monoclonal antibody directed against RANKL, a molecule which belongs to the tumour necrosis factor alpha (TNF-α) superfamily and exerts a pivotal role in modulating bone remodelling; denosumab binds RANKL with high affinity and specificity thus preventing interaction with its receptor on osteoclasts and their precursors. Consequently, mimicking OPG effect, it inhibits their differentiation, activation and, lastly, survival [78, 79]. In several clinical trials, denosumab showed to be more effective in reducing the risk of vertebral and non-vertebral fractures (including femoral ones) than the other antiresorptive drugs.

The ABSG-18 study randomized 3425 HR+ postmenopausal early BC patients to receive AI plus denosumab or AI plus placebo. Results showed that denosumab could delay the onset of fracture events and decrease their incidence when compared to the control arm (5% vs 9.6%). Denosumab also increased BMD at the total lumbar spine, total hip and femoral neck. The observed bone-protective effect was also reported with respect to the incidence of new and the worsening of pre-existing VFs; however, after denosumab discontinuation, the benefit was not maintained with accelerated bone loss [80].

Guidelines and recommendations agree that bone loss should be monitored and an antiresorptive intervention considered in patients with BMD decreases during AI-therapy [36•].

#### Antiresorptive drugs and survival outcomes

During the latest years, several clinical trials have also explored the impact of antiresorptive drugs on survival outcomes in HR+ BC patients undergoing adjuvant ET.

Regarding bisphosphonates, HOBOE-2 trial showed that the addition of zoledronate (4 mg intravenously every 6 months) to letrozole in premenopausal HR+ early BC patients determined a longer DFS compared to those treated with tamoxifen alone (gain of +8% after 5 years). However, the absence of a statistically significant difference in DFS between letrozole plus zoledronate versus letrozole alone arm could suggest that the previous result is probably due to different ET, rather than the addition of zoledronate [81•]. Moreover, premenopausal women enrolled in the Early Breast Cancer Trialists' Collaborative Group (EBCTCG) meta-analysis did not benefit from the addition of bisphosphonates neither in terms of bone recurrences (*p*=0.42) nor in terms of breast cancer–specific survival (*p*=0.96); on the other hand, postmenopausal patients achieved statistically significant reduction in both outcomes (−28%, *p*=0.0002, and −18%, *p*=0.002, respectively) [82].

Data reported on denosumab are also conflicting: in the aforementioned ABSG-18 trial, a DFS advantage at 8 years was observed by adding adjuvant denosumab (60 mg subcutaneously every 6 months) to AI alone (80% vs 77.5%, respectively) [80]. However, recent D-CARE study, in which BC patients, irrespective from menopausal status as well as HR and HER2 expression, were randomized to 5 years of adjuvant denosumab or placebo, did not show an advantage in terms of bone metastasis–free survival from the addition of denosumab, without differences among all analysed subgroups [83•].

Mechanisms underlying these controversial results in terms of DFS benefit reported in the aforementioned trials are still unclear, and several hypotheses have been proposed. The most intriguing one regards the presence of bone undetectable micro-metastases established by circulating tumour cells that colonize the osteoblastic niche [84]. These BC micro-metastases can remain quiescent potentially for many years, until, for reasons that are not well understood, they exit their dormancy status and start to proliferate, thus generating macro-metastases in the bone or elsewhere [85]. Being bisphosphonates as well as denosumab active on bone cells and T cell function, they could counteract this event by inducing bone microenvironment modifications that may be lethal for isolated cancer cells [86]. Finally, as to adjuvant denosumab, it is noteworthy mentioning the possible activity of anti-RANK molecules on the immunosuppressive effect exerted by the RANK–RANKL axis in antitumour immunity as pointed out by recent studies, suggesting that another potential mechanism of action of adjuvant anti-RANKL molecule could be the eradication of micrometastases through acquired immune activation [87–89]. Finally, the absence of survival effect in D-CARE trial and in the premenopausal cohort of EBCTCG metanalysis underlines a more complex scenario, in which probably other factors are involved in bone progression, especially among younger and high-risk BC patients.

#### Antiresorptive drugs: safety and treatment duration

Antiresorptive drugs are well tolerated, and their use in preventing CTIBL is safe. Class-specific adverse events (AEs) are well-known, and they can be optimally managed in clinical practice in order to avoid treatment discontinuation and obtain the maximum expected benefit. Bisphosphonates’ tolerability is strictly dependent from the way of administration. The irritative effect on upper gastrointestinal (GI) mucosa results into dyspepsia, nausea, vomiting, epigastric pain and esophagitis; for the last one, which have been typically reported during alendronate administration, real-life experiences led to recommend to swallow it with 180–240 ml of water in the morning, remaining upright (sitting, standing or walking) for up to an hour afterwards, discontinuing the drug if these measures fail; a proper administration of oral bisphosphonates could minimize upper GI symptoms [90, 91]. Intravenous bisphosphonates have been linked to renal toxicity, namely toxic acute tubular necrosis, which is higher in older patients with pre-existent chronic kidney disease, thus suggesting to calculate creatinine clearance in patients before starting treatment [90]. Acute phase reaction, which could affect up to 70% of bisphosphonates-naïve patients treated with the first dose of intravenous zoledronate (less frequently with ibandronate and subsequent doses), consists of transient flu-like symptoms (myalgia or arthralgia, fatigue, pyrexia, nausea, headache), and could be severe even if it does not generally require drug discontinuation [92]. This reaction, which has not been observed with non-nitrogen-containing bisphosphonates, could also be a rare consequence of oral administration [90].

Denosumab at the dose commonly used for treating osteoporosis is safe, as shown in clinical trials and post-marketing surveillance results, being musculoskeletal pain the most frequent adverse event [93]; notably, no increase in the risk of cancer, delayed fracture healing or serious AEs was reported compared to placebo [93, 94].

Common AEs of antiresorptive drugs are medication-related osteonecrosis of the jaw (MRONJ) and hypocalcemia. MRONJ onset, which was described for the first time in early 2000s in patients treated with antiresorptive agents, could be favoured by additional risk factors such as the use of corticosteroids, smoking habit and comorbidities (i.e. diabetes mellitus, immunological disorders). In order to prevent MRONJ, good oral hygiene must be maintained and invasive dental procedures must be absolutely avoided [95], and, before starting treatment, a dental visit is recommended with extraction of teeth and implants that are beyond salvaging [96]. Nowadays, the incidence of these AEs seems to be very low with both bisphosphonates and denosumab, administered at their recommended schedules [95, 97].

The optimal duration of antiresorptive treatments during adjuvant ET is not well defined; however, all the main international guidelines recommend their administration for the whole duration of the adjuvant ET [98, 99•, 100]. Data supporting this recommendation are indirect and based on pivotal studies performed on patients treated with AIs, where the concomitant use of antiresorptive agents appeared to reduce the risk of fractures and preserve the BMD by modulating bone turnover; nevertheless, the hypothesis of bone pre-metastatic niches regulated by RANK/RANKL axis has been disproved by results of the recent D-CARE trial, in which 5 years of adjuvant denosumab in high-risk stage II/III BC patients, independently from hormonal status, did not improve bone metastasis–free survival [83•], thus questioning the potential anti-cancer activity of anti-RANKL in earlier phases, without regard of duration of treatment.

## Conclusions

Adjuvant ET is a keystone of treatment for HR+ early BC. As a consequence, CTIBL represents the most common long-term adverse event experienced by BC patients. Early detection and proper management of CTIBL, especially in patients treated with AIs, are crucial in preventing risk fracture in order to simultaneously improve quality of life.

Non-pharmacological interventions (such as lifestyle changes) as well as the administration of antiresorptive drugs (bisphosphonates and denosumab) should be considered for BC patients on adjuvant AI-therapy, as recommended by international guidelines.

Further clinical trials as well as increased awareness of CTIBL among clinicians are needed to improve the management of this common long-term adverse event of BC treatment.
